# Denoising self-supervised learning for disease-gene association prediction

**DOI:** 10.1186/s12859-025-06281-3

**Published:** 2025-10-23

**Authors:** Yan Zhang, Ju Xiang, Jianming Li

**Affiliations:** 1 School of Computer Science and Engineering, Central South University, Changsha, China; 2 School of Computer Science and Engineering, Hunan University of Information Technology, Changsha, China; 3 School of Computer Science and Technology, Changsha University of Science and Technology, Changsha, China; 4 Human Provincial University Key Laboratory of the Fundamental and Clinical Research on Neurodegenerative Diseases, Changsha Medical University, Changsha, China

**Keywords:** Disease-gene associations prediction, Similarity-guided, Denoising self-supervised learning

## Abstract

Understanding the interplay between diseases and genes is crucial for gaining deeper insights into disease mechanisms and optimizing therapeutic strategies. In recent years, various computational methods have been developed to uncover potential disease-gene associations. However, existing computational approaches for disease-gene association prediction still face two major limitations. First, most current studies focus on constructing complex heterogeneous graphs using multi-dimensional biological entity relationships, while overlooking critical latent interaction patterns, namely, disease neighbor interactions and gene neighbor interactions—which are more valuable for association prediction. Second, in self-supervised learning (SSL), the presence of noise in auxiliary tasks commonly affects the accurate modeling of diseases and genes. In this study, we propose a novel denoising method for disease-gene association prediction, termed DGSL. To address the first issue, we utilize bipartite graphs corresponding to diseases and genes to derive disease-disease and gene-gene similarities, and further construct disease and gene interaction graphs to capture the latent interaction patterns. To tackle the second challenge, we implement cross-view denoising through adaptive semantic alignment in the embedding space, while preserving useful neighbor interactions. Extensive experiments on benchmark datasets demonstrate the effectiveness of our method.

**Supplementary Information:**

The online version contains supplementary material available at 10.1186/s12859-025-06281-3.

## Introduction

The completion of the Human Genome Project has opened the door to new research opportunities and challenges. Identifying gene-disease associations can facilitate the development of new technologies for disease prevention, diagnosis, and treatment [28]. Therefore, accelerating the discovery of associations between diseases and genes is beneficial for advancing disease prevention and treatment. Earlier studies have made great efforts to achieve this goal, such as automated literature mining based on prior knowledge [28] and scanning phenotypic data to identify genetic associations [4]. Despite their effectiveness, these approaches are labor-intensive and time-consuming, which hinders their large-scale application. More importantly, they rely heavily on specialized expertise, raising the technical barrier for researchers.

With the rapid advancement of computer science, computational approaches have been employed to identify potential disease-related genes from large-scale data. Li et al. constructed a heterogeneous network using phenotype-gene relationships from the OMIM database and extended the random walk with restart algorithm to heterogeneous networks to uncover gene-phenotype associations and reveal hidden disease relationships [22]. Lee et al. explicitly modeled the uncertainty of associations and incorporated this uncertainty into the "guilt by association" (GBA) framework to enable confident identification of disease-related genes [18]. Singh-Blom et al. proposed a method called Katz measure to predict gene-disease associations in model organisms, inspired by its success in social network link prediction [32]. In addition, other similar studies have been conducted, and these approaches are collectively referred to as network-based computational methods [6, 8, 23, 40]. Although such methods align well with the intrinsic nature of biological networks, they are limited in handling noisy input data and often suffer from poor generalization capability.

In addition to network-based approaches, a few machine learning methods have also been explored for disease-gene association prediction. For example, Mordelet et al. proposed a novel algorithm called ProDiGe for disease gene prioritization [27], and Yang et al. developed an algorithm named PUDI, which can identify disease-associated genes with higher accuracy [41]. However, due to the limited feature representation capacity and scalability issues, these traditional machine learning methods have gradually given way to more powerful deep learning-based approaches. Deep learning methods, due to their powerful representation learning capabilities and strong generalization performance, have been applied to various tasks in bioinformatics and biomedical entity association prediction [25, 36, 39, 42]—including, of course, disease-gene association prediction. In related areas, deep learning has also been integrated with single-cell RNA sequencing (scRNA-seq) data to reconstruct gene regulatory networks while addressing noise reduction and class imbalance challenges [44]. Likewise, in drug discovery, advanced graph-based frameworks have been proposed to integrate local and global node features for accurate drug–protein interaction prediction [43]. These studies highlight the versatility of graph learning and anomaly detection techniques in capturing complex biological relationships, which further motivate our investigation into disease–gene association prediction. Han et al. proposed a novel framework for disease-gene association prediction, called GCN-MF, which integrates graph convolutional networks (GCN) with matrix factorization [7]. Wang et al. considered the biological annotations of genes and employed a hypergraph-based embedding technique to extract deep features underlying the annotations [34]. Li et al. introduced DGP-PGTN, an end-to-end disease-gene association prediction model based on a parallel graph transformer network, which deeply integrates heterogeneous information from diseases, genes, ontologies, and phenotypes [21]. Wang et al. constructed a biomedical knowledge graph centered on diseases and genes and developed an end-to-end knowledge graph completion framework for disease-gene prediction named KDGene, based on interactive tensor decomposition, to enhance predictive accuracy [35]. These studies, including [11, 26, 37], focus on modeling complex multi-entity heterogeneous graphs, which may lead to mixed processing of different semantic paths and limit the expressive capacity of the models. Some works have directly or indirectly employed the concept of meta-paths [3, 9, 24] by predefining paths to explicitly specify semantic relationships in the network, they help reduce redundant connections and enhance the model's focus on meaningful associations.

Although these deep learning-based methods have achieved promising results, most of them overlook the fundamental interaction patterns underlying disease-gene association prediction—namely, disease neighborhood interactions and gene neighborhood interactions. To address this issue, Xie et al. proposed a graph mutual information maximization layer to maximize the mutual information between a node and its neighbors [38]. Moreover, the self-supervised learning paradigm, as an effective strategy to alleviate data sparsity, has also been applied to disease-gene association prediction tasks [31, 38]. However, the neighborhood information used in self-supervised learning (as auxiliary tasks) inevitably introduces noise.

In this study, we propose a novel denoising self-supervised learning method, DGSL, for disease-gene association prediction, aiming to uncover neighborhood interactions and alleviate noise inherent in self-supervised learning. First, during the data preprocessing stage, we derive disease-disease and gene–gene similarities from bipartite graphs corresponding to diseases and genes, respectively, and subsequently construct disease and gene interaction graphs. This simple yet effective approach captures underlying interaction patterns, enabling more comprehensive representations of diseases and genes. Second, we introduce a denoising self-supervised learning paradigm that performs cross-view denoising via adaptive semantic alignment in the embedding space, while preserving informative interaction signals. Extensive experiments on benchmark dataset demonstrate the robustness of our model in handling data sparsity and noise, as well as its effectiveness in improving prediction accuracy. Case studies further validate the effectiveness of our proposed similarity-guided neighborhood interaction mining and denoising self-supervised learning approach.

## Materials and methods

### Dataset

#### Raw data

We use the dataset collected by He et al., which includes four different types of nodes: genes, diseases, gene ontologies (GO), and disease symptoms [9]. Specifically, they collected 130,820 disease-gene associations from DisGeNet, 99,087 disease-symptom associations from HPO and Orphanet, and 218,337 annotation records from STRING 10. Detailed information is shown in Table 1.

**Table 1:** Statistical information of benchmark dataset

Node	Number	Associations	Number
Gene	21,584	Disease-gene	130,820
Disease	15,030	Disease-symptom	99,087
GO	14,204	Gene-GO	218,337
Symptom	6,540		

#### Preprocessed data

Based on the raw data, we obtained the dataset actually used in our study through a preprocessing procedure. First, we used the 130,820 disease-gene associations collected from DisGeNet to extract 13,074 diseases and 8,947 genes that are actually involved in the associations. Then, we used the corresponding disease-symptom associations and gene–gene ontology associations to derive disease–disease similarity and gene–gene similarity. Taking the similarity computation between disease ${d}_{i}$ and disease ${d}_{j}$ as an example, the process is as follows:

1 $$sim_{i,j} = \frac{{2\left| {\left\{ {t_{1} |t_{1} \in N_{{d_{i} }} } \right\} \cap \left\{ {t_{2} |t_{2} \in N_{{d_{j} }} } \right\}} \right|}}{{\left| {\left\{ {t_{1} |t_{1} \in N_{{d_{i} }} } \right\}} \right| + \left| {\left\{ {t_{2} |t_{2} \in N_{{d_{j} }} } \right\}} \right|}}$$

where ${N}_{{d}_{i}}$ and ${N}_{{d}_{j}}$ represent the sets of symptoms associated with ${d}_{i}$ and ${d}_{j}$, respectively. Similarity, gene–gene similarity can also be indirectly derived from gene–gene ontology association data.

### Problem formulation

Diseases and genes can be represented as $D=\{{d}_{1},\cdots ,{d}_{m}\}$ and $G=\{{g}_{1},\cdots ,{g}_{n}\}$, respectively, where $m$ and $n$ denote the number of diseases and genes. The disease–gene interaction data can be represented by an association matrix $A$, where an entry is set to 1 if there exists an interaction between disease ${d}_{i}$ and gene ${g}_{j}$. To capture neighborhood information of diseases and genes, we utilize similarity information to construct a disease neighbor matrix ${A}_{d}$ and a gene neighbor matrix ${A}_{g}$, respectively. The goal of disease–gene association prediction is to learn a model based on the disease–gene association matrix $A$, in combination with the disease–disease neighbor matrix ${A}_{d}$ and the gene–gene neighbor matrix ${A}_{g}$, to predict potential associations between diseases and genes.

**Fig. 1:**
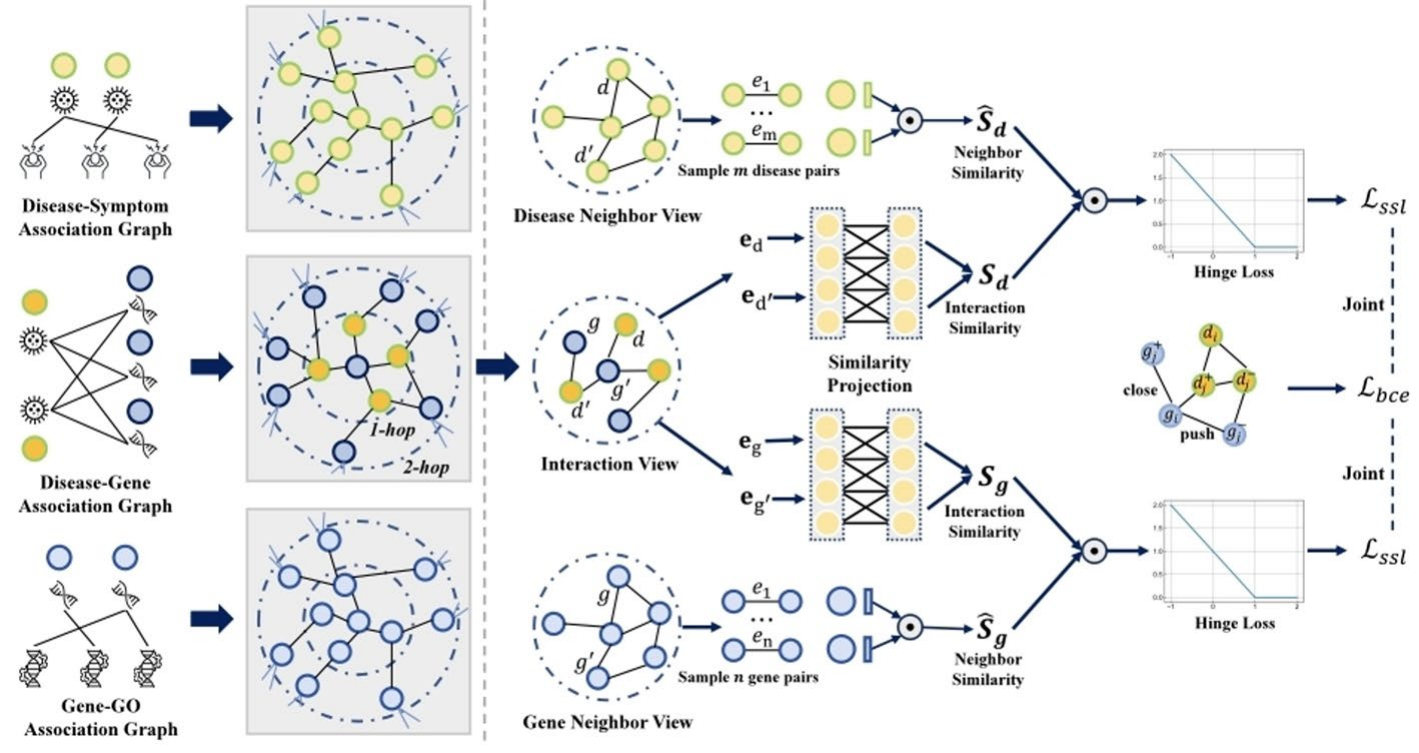
Overall framework of the proposed DGSL model. The overall framework of the DGSL model consists of three modules: (1) a relation extraction module, which is responsible for constructing association and neighborhood information; (2) a graph relation learning module, which leverages structural information from different views to perform message propagation and generate node representations; (3) a multi-task optimization module, which performs denoising self-supervised learning and jointly optimizes the model with the main loss

The model architecture is illustrated in Fig. 1.

### Cross-view graph relation learning

We first initialize the embeddings for diseases and genes and then employ cross-view modeling to capture the collaborative relationships among disease–gene associations, disease–disease associations, and gene–gene associations. Inspired by [10], we adopt a graph neural network without feature transformation and non-linear activation, which is formulated as follows:

2 $${\mathbf{E}}^{\left( l \right)} = \left( {\tilde{A} + {\mathbf{I}}} \right){\mathbf{E}}^{{\left( {l - 1} \right)}}$$

$$\begin{array}{ccc}{\mathbf{E}}^{(l)}& \in & {\mathbb{R}}^{(m+n)\times d}\end{array}$$ denotes the embeddings of diseases and genes after $l$ iterations of disease–gene association modeling. ${\mathbf{E}}^{(0)}$ represents the concatenation of the initial disease embeddings ${\mathbf{E}}_{d}$ and the initial gene embeddings ${\mathbf{E}}_{g}$. $\mathbf{I}$ denotes the identity matrix used for implementing self-loops, and $\widetilde{A}$ represents the Laplacian matrix of the disease–gene interaction matrix:

3 $$\tilde{A} = \left( {D^{{ - \frac{1}{2}}} \overline{A}D^{{ - \frac{1}{2}}} } \right), \overline{A} = \left[ {\begin{array}{*{20}c} 0 & A \\ {A^{{ \top }} } & 0 \\ \end{array} } \right]$$

$A$ denotes the disease-gene association matrix, $\overline{A }$ is the bidirectional adjacency matrix of the disease-gene association view, and $D$ is the diagonal degree matrix.

To leverage the latent neighborhood information of diseases and genes, we apply LightGCN to the disease-disease neighbor matrix ${A}_{d}$ to encode disease neighborhood information. We set ${\mathbf{E}}_{d}^{(0)}={\mathbf{E}}_{d}$, using the initial disease embeddings as input, and update the disease embeddings through multi-layer propagation:

4 $${\mathbf{E}}_{d}^{\left( l \right)} = \left( {\tilde{A}_{d} + {\mathbf{I}}_{d} } \right){\mathbf{E}}_{d}^{{\left( {l - 1} \right)}} , \tilde{A}_{d} = \left( {D_{d}^{{ - \frac{1}{2}}} A_{d} D_{d}^{{ - \frac{1}{2}}} } \right)$$

Here, ${\mathbf{E}}_{d}^{\left(l\right)}$ and ${\mathbf{E}}_{d}^{\left(l-1\right)}$ represent the disease embeddings at the $l$-th and $\left(l-1\right)$-th iterations, respectively. The disease-disease neighbor matrix ${A}_{d}$ is obtained using $top$-$k$ similarity:

5 $$A_{d}^{{\left( {i,j} \right)}} = \left\{ {\begin{array}{*{20}l} {1,} \hfill & {{\text{sim}}_{{i,j}} {\text{ belongs to the Top-k values of node }}i} \hfill \\ {0,} \hfill & {{\text{others}}} \hfill \\ \end{array} } \right.$$

Here, to reduce redundancy, the node itself is not selected as one of its neighbors. Analogously, we apply LightGCN to the gene–gene neighbor matrix ${A}_{g}$ to encode gene neighborhood information and finally obtain the updated gene embeddings ${\mathbf{E}}_{g}$ after the iteration.

We use mean pooling to obtain the final embeddings after iteration for each view:

6 $$\begin{array}{*{20}c} {{\overline{\mathbf{E}}} = \mathop \sum \limits_{l = 0}^{L} {\mathbf{E}}^{\left( l \right)} , {\overline{\mathbf{E}}}_{d} = \mathop \sum \limits_{l = 0}^{L} {\mathbf{E}}_{d}^{\left( l \right)} , {\overline{\mathbf{E}}}_{g} = \mathop \sum \limits_{l = 0}^{L} {\mathbf{E}}_{g}^{\left( l \right)} } \\ \end{array}$$

Then, the final embeddings representing diseases and genes are obtained by simply summing the embeddings from different views:

7 $${\mathbf{E}}_{d}^{f} = {\overline{\mathbf{E}}}_{{d^{\prime}}} + {\overline{\mathbf{E}}}_{d} ,{\mathbf{E}}_{g}^{f} = {\overline{\mathbf{E}}}_{{g^{\prime}}} + {\overline{\mathbf{E}}}_{g}$$

Here, ${\overline{\mathbf{E}} }_{{d}^{{^{\prime}}}}$ and ${\overline{\mathbf{E}} }_{{g}^{{^{\prime}}}}$ are the disease and gene embeddings split from $\overline{\mathbf{E} }$.

### Cross-View Semantic Alignment

Since our neighborhood information is constructed under similarity guidance, it inevitably contains noise. Inspired by [33], we introduce a cross-view denoising self-supervised learning paradigm to mitigate the noise introduced during the transfer of neighborhood information to the disease-gene association data, enabling unbiased self-supervised learning. Specifically, we design a learnable similarity projection function to map the disease-gene association semantics into a latent embedding space, as shown below:

8 $$s_{{m,m^{^\prime}}} = {\text{sigmoid}}\left( {{\mathbf{d}}^{{ \top }} \cdot \sigma \left( {{\mathbf{T}} \cdot \left[ {{\overline{\mathbf{e}}}_{m}^{{\left( {d^{^\prime}} \right)}} ;{\overline{\mathbf{e}}}_{{m^{^\prime}}}^{{\left( {d^{^\prime}} \right)}} } \right] + {\overline{\mathbf{e}}}_{m}^{{\left( {d^{^\prime}} \right)}} + {\overline{\mathbf{e}}}_{{m^{^\prime}}}^{{\left( {d^{^\prime}} \right)}} + {\mathbf{c}}} \right)} \right)$$

Here, ${s}_{m,{m}^{{^{\prime}}}}$ denotes the semantic similarity of a randomly sampled disease pair $\left(m,{m}^{{^{\prime}}}\right)$ in the disease-gene association view. ${\overline{\mathbf{e}} }_{m}^{\left({d}^{{^{\prime}}}\right)}$ and ${\overline{\mathbf{e}} }_{{m}^{{^{\prime}}}}^{\left({d}^{{^{\prime}}}\right)}$ represent the embeddings of disease $m$ and disease ${m}^{{^{\prime}}}$ under the disease-gene association view, obtained from ${\overline{\mathbf{E}} }_{{d}^{{^{\prime}}}}$. $\sigma \left(\cdot \right)$ denotes the LeakyReLU activation function, and $\mathbf{d}\in {\mathbb{R}}^{d}$,$\mathbf{T}\in {\mathbb{R}}^{d\times 2d}$, and $\mathbf{c}\in {\mathbb{R}}^{d}$ are learnable parameters in the projection function. The semantic similarity of the pair $\left(m,{m}^{{^{\prime}}}\right)$ in the disease neighborhood view can be simply represented as:${\hat{s}}_{m,{m}^{{^{\prime}}}}={\overline{e} }_{m}^{(d)\top }{\overline{e} }_{{m}^{{^{\prime}}}}^{(d)}$, ${\overline{\mathbf{e}} }_{m}^{(d)}$ and ${\overline{\mathbf{e}} }_{{m}^{{^{\prime}}}}^{(d)}$ are obtained from ${\overline{\mathbf{E}} }_{d}$. The semantic similarity ${s}_{n,{n}^{{^{\prime}}}}$ of a randomly sampled gene pair $\left(n,{n}^{{^{\prime}}}\right)$ in the disease-gene association view and the semantic similarity ${\widehat{s}}_{n,{n}^{{^{\prime}}}}$ in the gene neighborhood view are obtained in the same manner.

### Multi-task optimization

Given the disease embedding ${\mathbf{e}}_{d}^{f}$ and the gene embedding ${\mathbf{e}}_{g}^{f}$, we can compute the association probability of the disease-gene pair: $\hat{y}={e}_{d}^{f{\top }}{e}_{g}^{f}$. Subsequently, a weighted binary cross-entropy loss function is used as the main optimization objective:

9 $${\mathcal{L}}_{bce} = - \frac{1}{n \times m}\left( {\gamma \mathop \sum \limits_{{\left( {i,j} \right) \in S_{dg}^{ + } }} \log \left( {\hat{y}_{ij} } \right) + \mathop \sum \limits_{{\left( {i,j} \right) \in S_{dg}^{ - } }} \left( {1 - \log \left( {\hat{y}_{ij} } \right)} \right)} \right)$$

where $(i,j)$ denotes the pair of disease ${d}_{i}$ and gene ${g}_{j}$, ${S}_{dg}^{+}$ denotes the set of all known disease-gene association pairs, and ${S}_{dg}^{-}$ represents the set of all unknown or unseen disease-gene association pairs. The balance factor $\gamma =\frac{\left|{S}_{dg}^{-}\right|}{\left|{S}_{dg}^{+}\right|}$ emphasizes the importance of observed associations to mitigate the damage of data imbalance, where $\left|{S}_{dg}^{-}\right|$ and $\left|{S}_{dg}^{+}\right|$ are the number of pairs in ${S}_{dg}^{-}$ and ${S}_{dg}^{+}$.

In addition, we employ a self-supervised learning task for cross-view alignment. Specifically, the cross-view alignment loss functions for diseases and genes are defined as follows:

10 $${\mathcal{L}}_{ssl}^{d} = \mathop \sum \limits_{{\begin{array}{*{20}c} {\left( {d_{m} ,d_{{m^{^\prime}}} } \right)} \\ \end{array} }} max\left( {0,1 - s_{{m,m^{^\prime}}} \hat{s}_{{m,m^{^\prime}}} } \right)$$

11 $${\mathcal{L}}_{ssl}^{g} = \mathop \sum \limits_{{\begin{array}{*{20}c} {\left( {g_{n} ,g_{{n^{^\prime}}} } \right)} \\ \end{array} }} max\left( {0,1 - s_{{n,n^{^\prime}}} \hat{s}_{{n,n^{^\prime}}} } \right)$$

By treating the above self-supervised learning objective as an auxiliary task, the noise from the neighborhood can be mitigated, thereby enhancing the model’s predictive performance. Therefore, the overall optimization objective of the model is:

12 $${\mathcal{L}} = {\mathcal{L}}_{bce} + \alpha \left( {{\mathcal{L}}_{ssl}^{d} + {\mathcal{L}}_{ssl}^{g} } \right)$$

where $\alpha$ is a balancing factor.

## Experiments

### Experimental setup

#### Baselines

The comparative baselines used in this study are: dgn2vec [23], Katz [32], RWRH [22], node2vec [6], VGAE [17], NIMGSA [13], GCN [16], MiGCN [38], GlaHGCL [31], DGP-PGTN [21] and KDGene [35].

#### Evaluation metrics

This study employs a total of seven evaluation metrics to assess predictive performance, namely: Area Under the ROC Curve (AUROC), Area Under the Precision-Recall Curve (AUPRC), Accuracy, Precision, Recall, F1 Score, and Matthews Correlation Coefficient (MCC).

#### Implementation details

We adopt 10-fold cross-validation to evaluate performance and avoid bias caused by data imbalance. Specifically, the positive samples are evenly divided into ten parts, each of which is used as the test set in turn. Due to the severe imbalance between positive and negative samples, we randomly sample an equal number of negative samples in each fold to form the test set together with the positive samples.

The hyperparameter $k$ for constructing the neighbor matrix is empirically set to 10. The embedding dimension is set to 64, the number of sampled neighbor pairs is 64, and the balancing factor $\alpha$ is set to 0.01. All model parameters are initialized using Xavier initialization [5] and optimized with the Adam optimizer [15], with an initial learning rate of 0.01. The hyperparameters of the baseline models are strictly set according to the recommended values in the corresponding literature. All experiments, including the comparative studies, are conducted on a machine equipped with a single 24 GB Nvidia RTX 3090 GPU.

### 10-fold cross-validation

As shown in Table 2, our model achieves the best overall performance across all seven evaluation metrics. It achieves a well-balanced performance between precision and recall, leading to a superior overall result in terms of the F1 score. The improvement in MCC further demonstrates the model’s robustness in handling class imbalance. These results collectively indicate that the proposed DGSL model possesses stronger discriminative power and generalization ability in the task of disease-gene association prediction.

**Table 2:** Performance comparison under tenfold cross-validation. *indicates that DGSL significantly outperforms other methods with *p*-values < 0.05 using the paired t-test

Model	Evaluation Criteria						
	AUROC	AUPRC	Accuracy	Precision	Recall	F1	MCC
DGP-PGTN Katz NIMGSA RWRH dgn2vec MiGCN node2vec GlaHGCL KDGene GCN VGAE DGSL	0.5354 ± 0.001 0.6229 ± 0.002 0.7294 ± 0.003 0.7715 ± 0.006 0.7991 ± 0.002 0.8422 ± 0.001 0.8667 ± 0.002 0.9043 ± 0.002 0.9172 ± 0.002 0.9194 ± 0.003 0.9281 ± 0.001 0.9423 ± 0.002*	0.5269 ± 0.001 0.5755 ± 0.001 0.6463 ± 0.002 0.8028 ± 0.006 0.8248 ± 0.003 0.8497 ± 0.002 0.8891 ± 0.002 0.9233 ± 0.001 0.9103 ± 0.003 0.9291 ± 0.005 0.9282 ± 0.002 0.9521 ± 0.003*	0.5000 ± 0.000 0.5014 ± 0.001 0.5690 ± 0.002 0.5001 ± 0.006 0.7177 ± 0.002 0.5031 ± 0.003 0.6045 ± 0.002 0.7875 ± 0.003 0.8165 ± 0.002 0.8607 ± 0.004 0.7677 ± 0.002 0.8913 ± 0.003*	0.2500 ± 0.000 0.0186 ± 0.001 0.5401 ± 0.003 0.0070 ± 0.008 0.8640 ± 0.002 0.5025 ± 0.002 0.3726 ± 0.001 0.8667 ± 0.004 0.8717 ± 0.003 0.9199 ± 0.004 0.6960 ± 0.002 0.9275 ± 0.002*	0.5000 ± 0.000 0.5420 ± 0.002 0.9285 ± 0.004 0.5105 ± 0.008 0.5166 ± 0.004 **0.9864 ± 0.002** 0.6950 ± 0.001 0.6667 ± 0.003 0.7427 ± 0.005 0.7904 ± 0.004 0.9548 ± 0.004 0.8544 ± 0.005	0.3333 ± 0.000 0.0360 ± 0.001 0.6830 ± 0.004 0.0138 ± 0.006 0.6466 ± 0.003 0.6651 ± 0.004 0.4851 ± 0.001 0.7536 ± 0.002 0.8019 ± 0.003 0.8503 ± 0.004 0.8051 ± 0.003 0.8893 ± 0.003*	0.0000 ± 0.000 0.0111 ± 0.002 0.1986 ± 0.004 0.0017 ± 0.007 0.4754 ± 0.004 0.0250 ± 0.002 0.2360 ± 0.001 0.7080 ± 0.001 0.6400 ± 0.004 0.7282 ± 0.003 0.5764 ± 0.003 0.7896 ± 0.006*

The best result in each metric is highlighted in bold, and the second -best result is underlined

In addition, we introduced more modality information to construct the final disease and gene similarities, thereby improving biological fidelity and reducing bias. The data sources of the additional modalities are provided in Supplementary Table 1, and the similarity construction process is described in Supplementary Method 1. We also performed tenfold cross-validation of DGSL against five strong baseline models on the new dataset, as shown in Supplementary Table 2.

### Head-to-head comparison

**Fig. 2:**
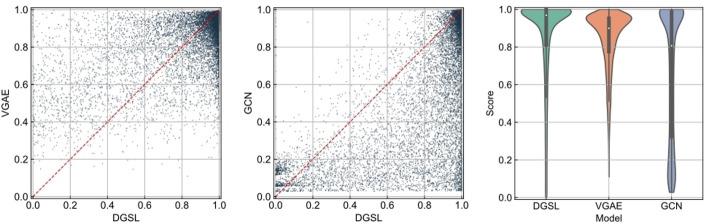
Head-to-head comparison of DGSL with VGAE and GCN

Disease-gene association prediction can be formulated as a binary classification task. Therefore, higher prediction scores for positive samples indicate that the model performs better in learning potential association features. Based on this, we conducted a head-to-head comparison between our model, DGSL, and the two baseline models with the best overall performance: VGAE and GCN. As shown in Fig. 2, each scatter point represents a positive sample, where the x-axis denotes the prediction score from DGSL and the y-axis denotes the score from the baseline model. We observe that most data points lie below the diagonal line. In addition, the violin plots in Fig. 2 provide a more intuitive visualization of the score distributions from different models. The median and quartiles of our model are higher than those of the baseline models, indicating that DGSL is more effective at capturing potential association features.

### Ablation studies

DGSL simultaneously incorporates disease neighborhood information and gene neighborhood information and introduces a denoising self-supervised learning strategy to mitigate both data sparsity and noise in the neighborhood information. Therefore, we design the following seven variants for ablation studies:

(i) **w/o disease**: Only considers gene neighborhood information, excluding disease neighborhood information. (ii)** w/o gene**: Only considers disease neighborhood information, excluding gene neighborhood information. (iii) **w/o both**: Neither disease nor gene neighborhood information is considered. (iv) **w/o** ${\mathcal{L}}_{{{s}}{{s}}{{l}}}^{{{d}}}$: Only the denoising self-supervised learning for genes is retained, while that for diseases is removed. (v) **w/o** ${\mathcal{L}}_{{{s}}{{s}}{{l}}}^{{{d}}}$: Only the denoising self-supervised learning for diseases is retained, while that for genes is removed. (vi) **w/o** ${\mathcal{L}}_{{{s}}{{s}}{{l}}}$: Neither the denoising self-supervised learning for diseases nor for genes is considered. (vii) **with CL**: Replaces the denoising self-supervised learning strategy with a conventional contrastive learning (CL) paradigm.

As shown in Table 3, all variants exhibit performance degradation across multiple metrics. The performance drop in the first three variants indicates that the neighborhood information we used has effectively enriched the representations of diseases and genes. When the denoising self-supervised learning is replaced with contrastive learning (CL), the performance declines significantly, suggesting that although CL has certain advantages in alleviating data sparsity, it lacks the ability to control noise in neighborhood information.

**Table 3:** Ablation study of DGSL under different settings

Model	Evaluation Criteria				
	AUROC	AUPRC	Accuracy	Precision	MCC
DGSL (entire model)	**0.942**	**0.952**	**0.891**	**0.927**	**0.789**
w/o disease	0.915	0.931	0.810	0.758	0.632
w/o gene	0.939	0.948	0.885	0.875	0.770
w/o both	0.908	0.930	0.750	0.685	0.533
w/o ${\mathcal{L}}_{ssl}^{d}$	0.921	0.935	0.845	0.819	0.692
w/o ${\mathcal{L}}_{ssl}^{g}$	0.939	0.951	0.889	0.919	0.783
w/o ${\mathcal{L}}_{ssl}$	0.934	0.949	0.832	0.781	0.674
with CL	0.939	0.947	0.826	0.769	0.666

The best result in each metric is highlighted in bold

### Robustness against noise

**Fig. 3:**
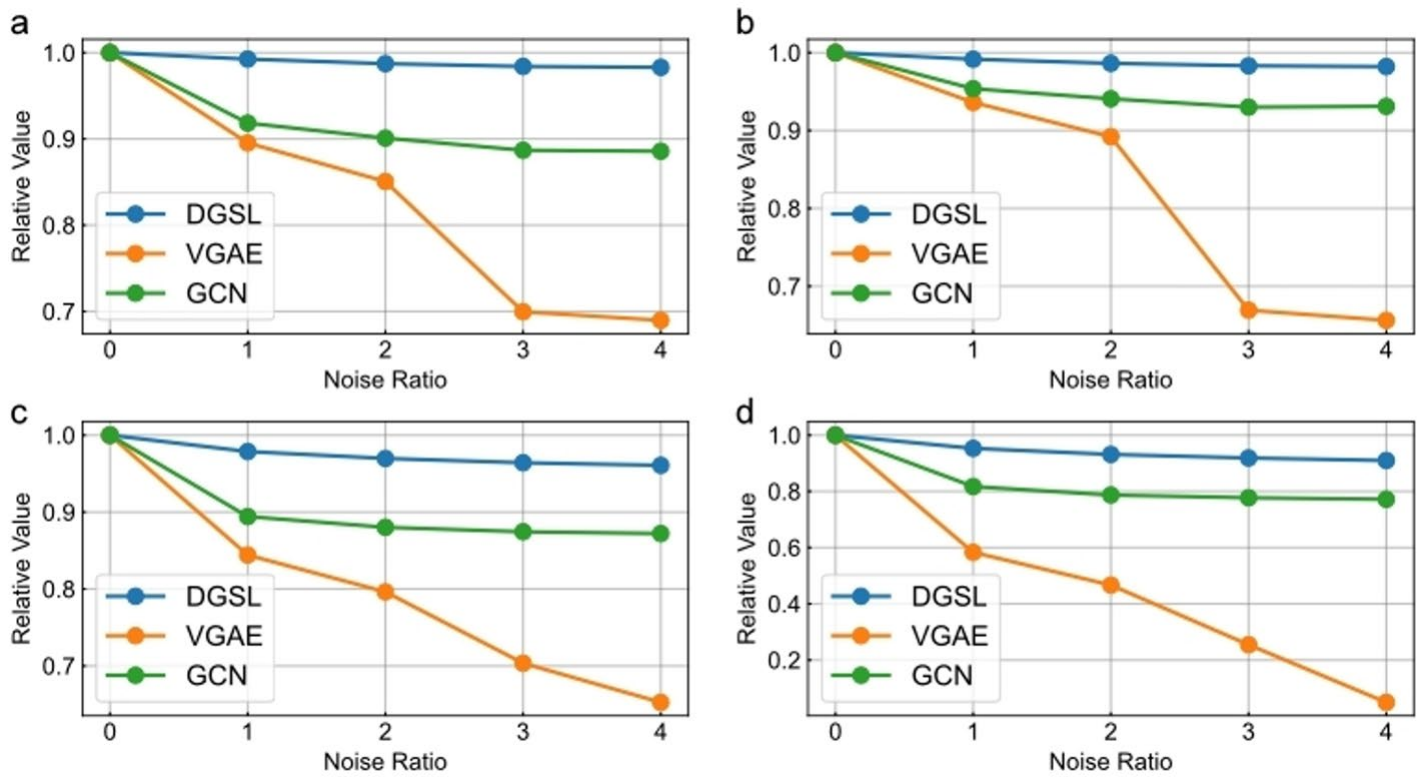
Performance variations of different models under varying levels of noise. **a**, **b**, **c**, and **d** represent the relative performance changes of different models under varying noise levels in terms of AUROC, AUPRC, Accuracy, and MCC, respectively

We add varying proportions of noise to the training data to observe changes in model performance. Specifically, we randomly insert pseudo-edges into the training data at ratios of 1x, 2x, 3x, and 4x elative to the number of true edges, and compare the relative performance of DGSL with that of VGAE and GCN. Here, relative performance refers to the ratio between the performance after adding pseudo-edges and the original performance. As shown in Fig. 3, the performance of our model remains stable across four evaluation metrics under different levels of noise, indicating the robustness of DGSL against noise.

### Robustness against data sparsity

**Fig. 4:**
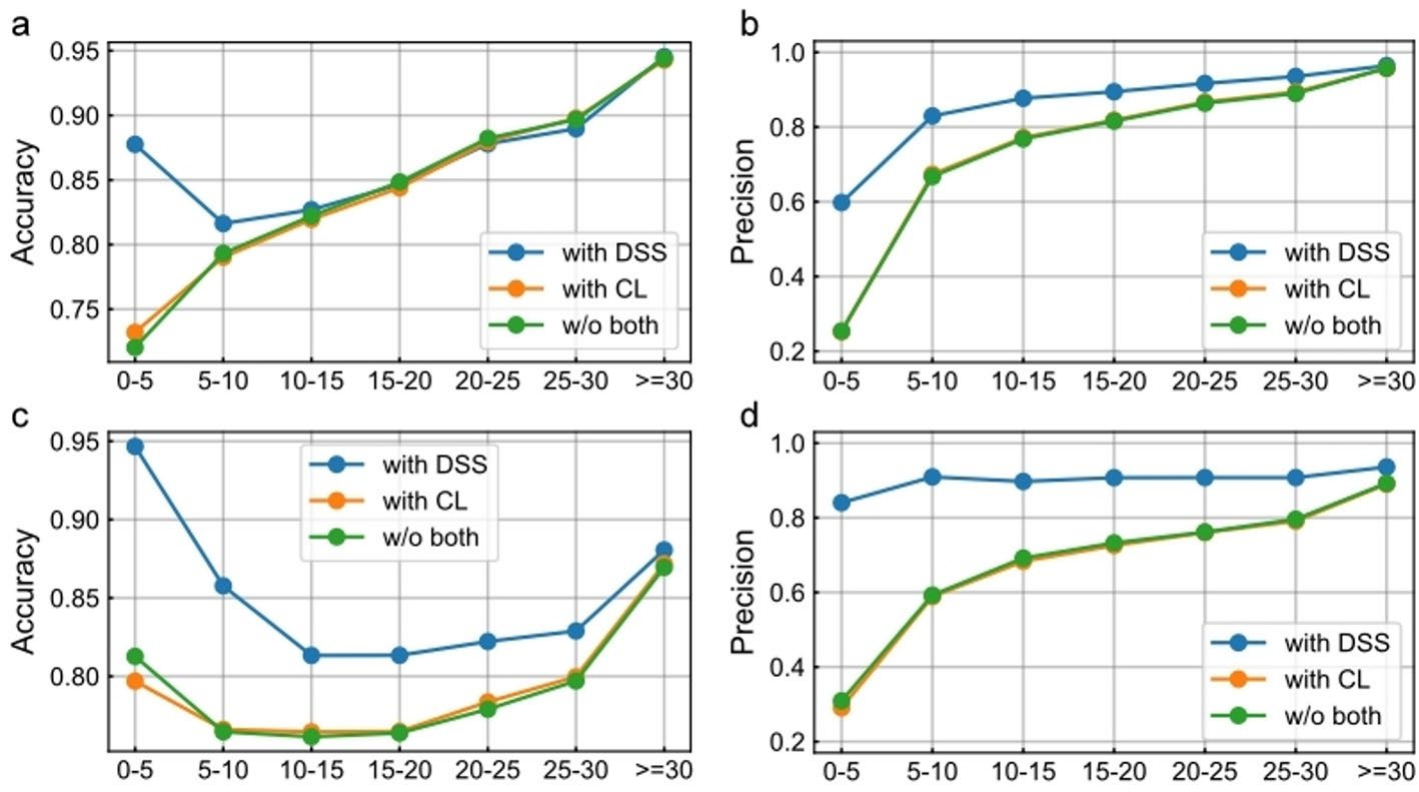
Performance of different models across groups, with varying levels of sparsity. **a** and **b** represent the Accuracy and Precision performance on the disease side, respectively, while **c** and **d** represent the Accuracy and Precision performance on the gene side, respectively

To verify the potential of our proposed denoising self-supervised learning (DSS) in addressing data sparsity, we compared DSS with contrastive learning (CL). Specifically, diseases and genes were grouped based on their number of associations, and the testing performance of each group was evaluated under tenfold cross-validation. As shown in Fig. 4, DSS consistently outperforms CL in terms of accuracy and precision.

**Fig. 5:**
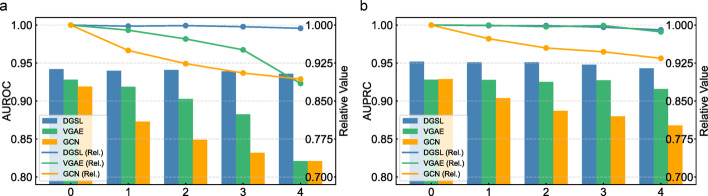
Performance of different models under varying levels of sparsity. In the x-axis, 1, 2, 3, and 4 represent randomly dropping 20%, 40%, 60%, and 80% of the edges from the training data, respectively. **a** and **b** correspond to the performance changes of different models in terms of AUROC and AUPRC, respectively. The bar charts and line graphs represent absolute and relative performance changes, corresponding to the left and right axes, respectively

To further validate the capability of DGSL in alleviating data sparsity, we randomly removed different proportions of edges from the training data and compared the relative performance with VGAE and GCN, as shown in Fig. 5. It can be observed that DGSL maintains stable performance across different levels of sparsity and consistently outperforms VGAE and GCN in terms of AUROC and AUPRC. These experimental results demonstrate the strong potential of DGSL in mitigating data sparsity.

### Cold-start experimental scenarios

In the disease-gene association prediction task, making accurate predictions for novel diseases or genes is crucial. Therefore, we designed a cold-start experiment to evaluate the predictive capability of DGSL on unseen diseases or genes. In this experimental setting, we randomly select a certain proportion of diseases or genes and remove all their known associations, using the remaining associations as the training set. The original associations of these held-out diseases or genes, along with an equal number of negative samples sampled based on their known association counts, form the test set. To avoid bias, the diseases and genes selected for different proportions are the same for DGSL and VGAE. As shown in Table 4, under various cold-start settings, DGSL consistently outperforms VGAE across multiple evaluation metrics.

**Table 4:** Performance comparison between DGSL and VGAE under cold-start scenarios with different proportions

Proportion	Prediction Side	Model	Evaluation criteria				
			AUROC	AUPRC	Accuracy	Precision	MCC
10%	Disease Side	VGAE	0.414	0.475	0.451	0.414	0.109
		DGSL	**0.840**	**0.826**	**0.652**	**0.872**	**0.377**
	Gene Side	VGAE	0.525	0.581	0.500	0.501	0.001
		DGSL	**0.792**	**0.812**	**0.633**	**0.924**	**0.366**
20%	Disease Side	VGAE	0.400	0.473	0.436	0.407	0.132
		DGSL	**0.814**	**0.797**	**0.550**	**0.895**	**0.204**
	Gene Side	VGAE	0.502	0.579	0.495	0.508	0.007
		DGSL	**0.790**	**0.807**	**0.626**	**0.920**	**0.354**
30%	Disease Side	VGAE	0.374	0.462	0.412	0.377	0.179
		DGSL	**0.810**	**0.795**	**0.540**	**0.908**	**0.186**
	Gene Side	VGAE	0.443	0.532	0.453	0.476	0.086
		DGSL	**0.807**	**0.826**	**0.639**	**0.934**	**0.379**
40%	Disease Side	VGAE	0.409	0.500	0.431	0.418	0.135
		DGSL	**0.809**	**0.796**	**0.548**	**0.905**	**0.203**
	Gene Side	VGAE	0.493	0.583	0.491	0.533	0.010
		DGSL	**0.812**	**0.824**	**0.659**	**0.899**	**0.398**
50%	Disease Side	VGAE	0.419	0.520	0.449	0.458	0.090
		DGSL	**0.812**	**0.798**	**0.534**	**0.924**	**0.172**
	Gene Side	VGAE	0.471	0.575	0.478	0.537	0.033
		DGSL	**0.813**	**0.824**	**0.655**	**0.903**	**0.393**

The best result in each metric is highlighted in bold

### Case study

**Fig. 6:**
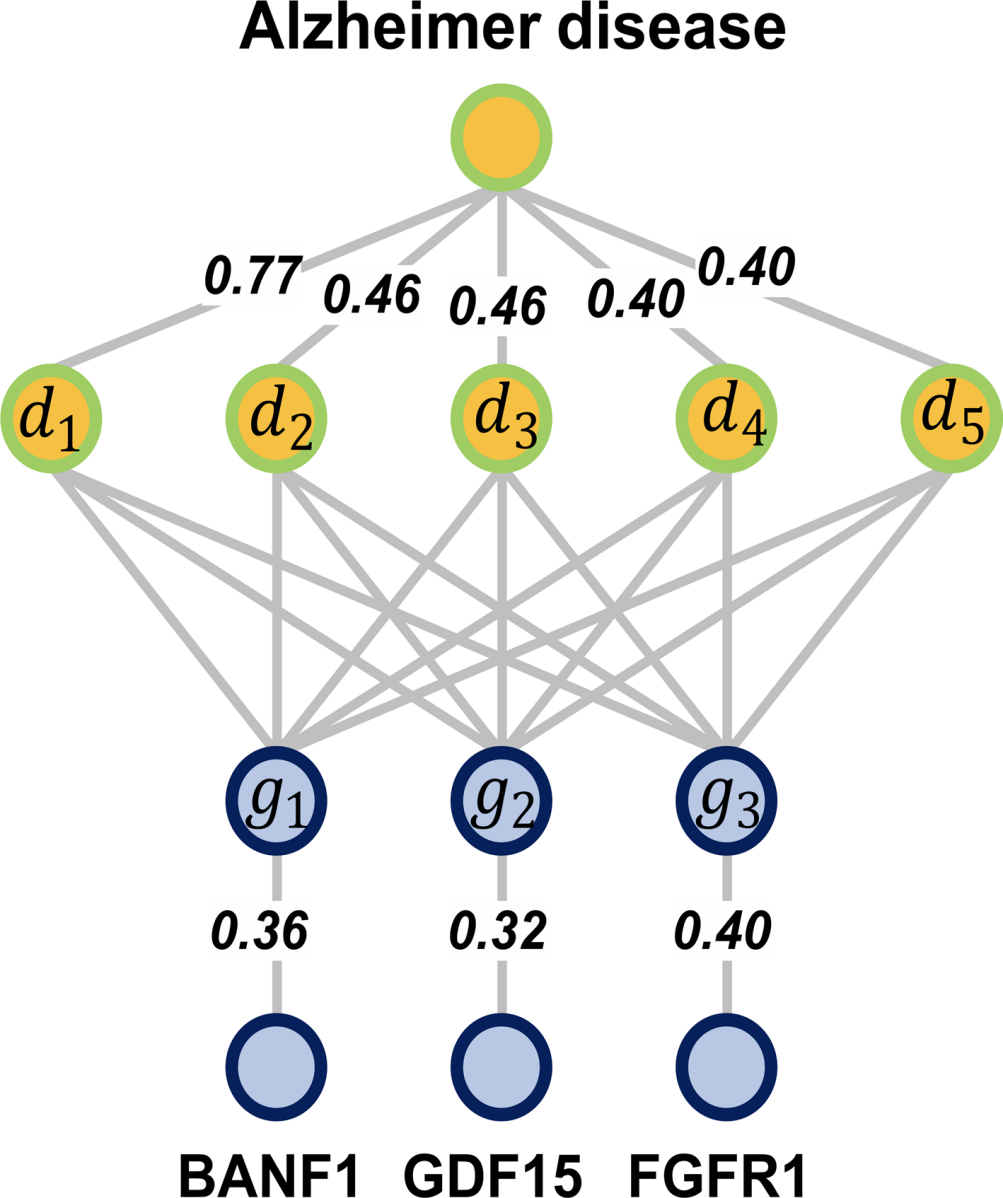
The 3-hop path graph for Alzheimer’s disease. *d*_1_ to *d*_5_ correspond to Alzheimer disease 2, Alzheimer disease 4, Lewy body dementia, Familial cancer of breast and Elliptocytosis 2, respectively, while *g*_1_ to *g*_2_ correspond to LMNA, GDF5, and FGFR2, respectively. The pairwise prediction scores between *d*_1_–*d*_5_ and *g*_1_–*g*_2_ are shown in Table 6

To evaluate the practical predictive capability of DGSL in real-world scenarios, we take Alzheimer’s disease as a case study and aim to predict its potential associated genes. Specifically, all known associations related to this disease are removed from the dataset, and the model is trained using the remaining associations. The top 10 candidate genes with the highest predicted scores are then identified for further investigation. As shown in Table 5, seven out of the top 10 candidate genes predicted by DGSL have been supported by existing studies. In addition, by integrating neighborhood similarity and model predictions, we successfully identified three potential genes that are indirectly associated with Alzheimer’s disease through 3-hop paths (as shown in Fig. 6), and these associations have also been supported by existing studies [2, 19, 29]. This demonstrates that our model, guided by similarity and enhanced by denoising self-supervised learning, can achieve interpretable and meaningful predictions in real-world scenarios.

**Table 5:** Top 10 associated genes of Alzheimer predicted by DGSL

Rank	Rank	Predicting scores	Supporting evidence
1	LMNA	0.988	[30]
2	GJA1	0.959	[14]
3	COL2A1	0.947	–
4	FGFR2	0.933	[1]
5	GDF5	0.932	–
6	LBR	0.93	[12]
7	CREBBP	0.901	–
8	HBB	0.899	[20]
9	FGFR3	0.896	[19]
10	ZMPSTE24	0.894	[30]

**Fig. 7:**
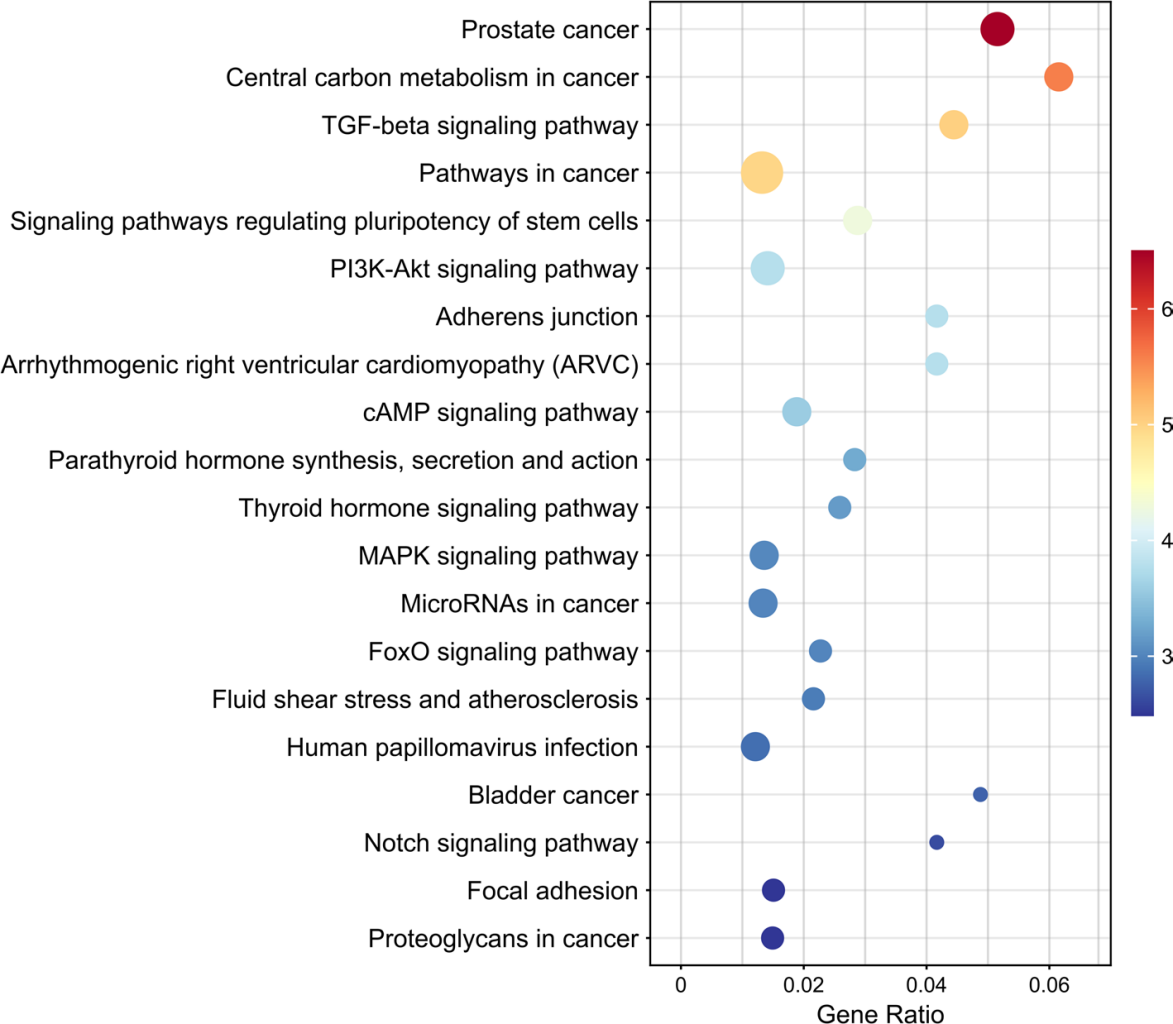
Pathway enrichment analysis of the top 30 candidate genes predicted by DGSL for Alzheimer’s disease. The information for these 30 genes is provided in Supplementary Table 3

In addition, we performed pathway enrichment analysis on the top 30 candidate genes predicted by DGSL for Alzheimer’s disease (AD), as shown in Fig. 7. The results indicate that among the top 20 enriched pathways, most are related to cancer or general metabolic and signaling pathways, such as “Prostate cancer” and “Pathways in cancer,” showing no obvious direct relevance to AD. However, several signaling pathways may play potential roles in the molecular mechanisms of AD. For example, the PI3K-Akt and MAPK signaling pathways can influence neuronal function and survival by regulating the phosphorylation status of tau protein, whose abnormal phosphorylation is a key factor in neurofibrillary tangle formation and neuronal degeneration in AD. The FoxO signaling pathway is involved in oxidative stress and apoptosis regulation, affecting neuronal viability; the Notch signaling pathway regulates neurodevelopment and post-injury repair, helping maintain neural network integrity; and the cAMP signaling pathway indirectly affects cognitive function by modulating synaptic plasticity and the expression of memory-related genes. Overall, these results suggest that although most enriched pathways are cancer-related, several key signaling pathways may intersect in AD pathogenesis through mechanisms involving tau protein regulation, oxidative stress, apoptosis, neural repair, and synaptic plasticity, providing potential leads for subsequent functional validation and mechanistic studies.

**Table 6:** The pairwise prediction scores between *d*_1_–*d*_5_ and *g*_1_–*g*_3_.

Disease	Gene	Predicting scores
Alzheimer disease 2 Alzheimer disease 2 Alzheimer disease 2 Alzheimer disease 4 Alzheimer disease 4 Alzheimer disease 4 Lewy body dementia Lewy body dementia Lewy body dementia Familial cancer of breast Familial cancer of breast Familial cancer of breast Elliptocytosis 2 Elliptocytosis 2 Elliptocytosis 2	LMNA GDF5 FGFR2 LMNA GDF5 FGFR2 LMNA GDF5 FGFR2 LMNA GDF5 FGFR2 LMNA GDF5 FGFR2	0.971 0.968 0.908 0.975 0.970 0.946 0.982 0.926 0.946 0.983 0.954 0.961 0.986 0.970 0.973

## Conclusion

In this study, we propose a denoising self-supervised learning framework, DGSL, for disease-gene association prediction. First, we employ a similarity-guided approach to effectively construct neighborhood information, enabling the model to focus more on the two key node types—diseases and genes—while obtaining more expressive representations. Furthermore, we introduce a denoising self-supervised learning strategy that mitigates noise in the neighborhood information through adaptive cross-view semantic alignment, while also enhancing the supervisory signal to alleviate data sparsity. Extensive experiments on benchmark datasets demonstrate the robustness and practicality of our model. Although DGSL achieves promising results, the current source of similarity information is relatively limited. In future work, we will explore more diverse network sources to enable a more comprehensive and scientifically grounded similarity-guided learning process.

## Supplementary Information

Below is the link to the electronic supplementary material.

Supplementary Material 1

## Data Availability

All data generated or analyzed during this study are included in this published article and its supplementary information files or are available from the corresponding author upon reasonable request.

## References

[1] Chen W, Wu L, Hu Y, Jiang L, Liang N, Chen J, et al. Microrna-107 ameliorates damage in a cell model of Alzheimer’s disease by mediating the FGF7/FGFR2/PI3K/Akt pathway. J Mol Neurosci. 2020;70(10):1589–97. 32472396 10.1007/s12031-020-01600-0

[2] Chiariello A, Valente S, Pasquinelli G, Baracca A, Sgarbi G, Solaini G, et al. The expression pattern of GDF15 in human brain changes during aging and in Alzheimer’s disease. Front Aging Neurosci. 2023;14:1058665. 36698863 10.3389/fnagi.2022.1058665 PMC9869280

[3] Cui W, Li S, Fang C, Long Q, Wang C, Wang X, et al. “Comprehensive metapath-based heterogeneous graph transformer for gene-disease association prediction”. In: 2024 IEEE International Conference on Bioinformatics and Biomedicine (BIBM): IEEE). 2024: 1463–1468.

[4] Denny JC, Ritchie MD, Basford MA, Pulley JM, Bastarache L, Brown-Gentry K, et al. PheWAS: demonstrating the feasibility of a phenome-wide scan to discover gene–disease associations. Bioinformatics. 2010;26(9):1205–10. 20335276 10.1093/bioinformatics/btq126 PMC2859132

[5] Glorot X, and Bengio Y. “Understanding the difficulty of training deep feedforward neural networks”. In: proceedings of the thirteenth international conference on artificial intelligence and statistics: JMLR workshop and conference proceedings. 2010: 249–256.

[6] Grover A, and Leskovec J. “node2vec: scalable feature learning for networks”. In: proceedings of the 22nd ACM SIGKDD international conference on knowledge discovery and data mining). 2016: 855–864. 10.1145/2939672.2939754 PMC5108654 27853626

[7] Han P, Yang P, Zhao P, Shang S, Liu Y, Zhou J, et al. “GCN-MF: disease-gene association identification by graph convolutional networks and matrix factorization”. In: proceedings of the 25th ACM SIGKDD international conference on knowledge discovery & data mining. 2019: 705–713.

[8] He B, Wang K, Xiang J, Bing P, Tang M, Tian G, et al. DGHNE: network enhancement-based method in identifying disease-causing genes through a heterogeneous biomedical network. Brief bioinfor. 2022;23(6):bbac405. 10.1093/bib/bbac405 36151744

[9] He M, Huang C, Liu B, Wang Y, Li J. Factor graph-aggregated heterogeneous network embedding for disease-gene association prediction. BMC Bioinformatics. 2021;22(1):165. 33781206 10.1186/s12859-021-04099-3 PMC8006390

[10] He X, Deng K, Wang X, Li Y, Zhang Y, and Wang M. “Lightgcn: Simplifying and powering graph convolution network for recommendation”. In: proceedings of the 43rd international ACM SIGIR conference on research and development in information retrieval. 2020: 639–648.

[11] Jia X, Luo W, Li J, Xing J, Sun H, Wu S, et al. A deep learning framework for predicting disease-gene associations with functional modules and graph augmentation. BMC Bioinfor. 2024;25(1):214. 10.1186/s12859-024-05841-3 PMC11549817 38877401

[12] Jiang L, Wolozin B. Oligomeric tau disrupts nuclear envelope via binding to lamin proteins and lamin B receptor. Alzheimers Dement. 2021;17:e054521.

[13] Jin C, Shi Z, Lin K, Zhang H. Predicting miRNA-disease association based on neural inductive matrix completion with graph autoencoders and self-attention mechanism. Biomolecules. 2022;12(1):64. 35053212 10.3390/biom12010064 PMC8774034

[14] Kajiwara Y, Wang E, Wang M, Sin WC, Brennand KJ, Schadt E, et al. GJA1 (connexin43) is a key regulator of Alzheimer’s disease pathogenesis. Acta Neuropathol Commun. 2018;6(1):144. 30577786 10.1186/s40478-018-0642-x PMC6303945

[15] Kinga, D., and Adam, J.B. “A method for stochastic optimization”. In: international conference on learning representations (ICLR): California;). 2015.

[16] Kipf T. Semi-supervised classification with graph convolutional networks. 2016. arXiv preprint arXiv:1609.02907.

[17] Kipf TN, and Welling M. Variational graph auto-encoders. 2016. arXiv preprint arXiv:1611.07308.

[18] Lee I, Blom UM, Wang PI, Shim JE, Marcotte EM. Prioritizing candidate disease genes by network-based boosting of genome-wide association data. Genome Res. 2011;21(7):1109–21. 21536720 10.1101/gr.118992.110 PMC3129253

[19] Li J-S, Yao Z-X. Modulation of FGF receptor signaling as an intervention and potential therapy for myelin breakdown in Alzheimer’s disease. Med Hypotheses. 2013;80(4):341–4. 23321060 10.1016/j.mehy.2012.12.008

[20] Li X, Tang P, Pang X, Song X, Xiong J, Yu L, et al. The features analysis of hemoglobin expression on visual information transmission pathway in early stage of Alzheimer’s disease. Sci Rep. 2024;14(1):15636. 38972885 10.1038/s41598-024-64099-0 PMC11228039

[21] Li Y, Guo Z, Wang K, Gao X, Wang G. End-to-end interpretable disease–gene association prediction. Brief Bioinform. 2023;24(3):bbad118. 36987781 10.1093/bib/bbad118

[22] Li Y, Patra JC. Genome-wide inferring gene–phenotype relationship by walking on the heterogeneous network. Bioinformatics. 2010;26(9):1219–24. 20215462 10.1093/bioinformatics/btq108

[23] Liu Y, Guo Y, Liu X, Wang C, Guo M. Pathogenic gene prediction based on network embedding. Brief Bioinform. 2021;22(4):bbaa353. 33367541 10.1093/bib/bbaa353

[24] Ma J, Qin T, Zhai M, Cai L. Agcnaf: predicting disease-gene associations using GCN and multi-head attention to fuse the similarity features. Eng Res Express. 2024;6(4):045221.

[25] Meng Y, Wang Y, Hu X, Lu C, Tang X, Cui F, et al. Adaptive debiasing learning for drug repositioning. J Biomed Inform. 2025. 10.1016/j.jbi.2025.104843. 40389101 10.1016/j.jbi.2025.104843

[26] Meng Z, Liu S, Liang S, Jani B, Meng Z. Heterogeneous biomedical entity representation learning for gene–disease association prediction. Brief Bioinform. 2024;25(5):bbae380. 39154194 10.1093/bib/bbae380 PMC11330343

[27] Mordelet F, Vert J-P. Prodige: prioritization of disease genes with multitask machine learning from positive and unlabeled examples. BMC Bioinformatics. 2011;12(1):389. 21977986 10.1186/1471-2105-12-389 PMC3215680

[28] Özgür A, Vu T, Erkan G, Radev DR. Identifying gene-disease associations using centrality on a literature mined gene-interaction network. Bioinformatics. 2008;24(13):i277–85. 18586725 10.1093/bioinformatics/btn182 PMC2718658

[29] Prissette M, Fury W, Koss M, Racioppi C, Fedorova D, Dragileva E, et al. Disruption of nuclear envelope integrity as a possible initiating event in tauopathies. Cell Rep. 2022. 10.1016/j.celrep.2022.111249. 36001963 10.1016/j.celrep.2022.111249

[30] Rosene MJ, Wen N, Li Z, Brase L, Hsu S, Cruchaga C, et al. LMNA-mediated nucleoskeleton dysregulation in Alzheimer disease. Alzheimers Dement. 2021;17:e054396.

[31] Si Y, Huang Z, Fang Z, Yuan Z, Huang Z, Li Y, et al. Global-local aware heterogeneous graph contrastive learning for multifaceted association prediction in miRNA–gene–disease networks. Brief Bioinform. 2024;25(5):bbae443. 39256197 10.1093/bib/bbae443 PMC11387071

[32] Singh-Blom UM, Natarajan N, Tewari A, Woods JO, Dhillon IS, Marcotte EM. Prediction and validation of gene-disease associations using methods inspired by social network analyses. PLoS ONE. 2013;8(5):e58977. 23650495 10.1371/journal.pone.0058977 PMC3641094

[33] Wang T, Xia L, and Huang, C. Denoised self-augmented learning for social recommendation. 2023. arXiv preprint arXiv:2305.12685.

[34] Wang T, Xu H, Zhang R, Xiao Y, Peng J, and Shang X. “Hypergraph-based gene ontology embedding for disease gene prediction”. In: 2022 IEEE international conference on bioinformatics and biomedicine (BIBM): IEEE). 2022: 2424–2430.

[35] Wang X, Yang K, Jia T, Gu F, Wang C, Xu K, et al. Kdgene: knowledge graph completion for disease gene prediction using interactional tensor decomposition. Brief Bioinform. 2024;25(3):bbae161. 38605639 10.1093/bib/bbae161 PMC11009469

[36] Wang Y, Meng Y, Zhou C, Tang X, Zeng P, Pan C, et al. Automatic collaborative learning for drug repositioning. Eng Appl Artif Intell. 2025;139:109653.

[37] Wang Z, Gu Y, Zheng S, Yang L, Li J. MGREL: a multi-graph representation learning-based ensemble learning method for gene-disease association prediction. Comput Biol Med. 2023;155:106642. 36805231 10.1016/j.compbiomed.2023.106642

[38] Xie J, Rao J, Xie J, Zhao H, Yang Y. Predicting disease-gene associations through self-supervised mutual infomax graph convolution network. Comput Biol Med. 2024;170:108048. 38310804 10.1016/j.compbiomed.2024.108048

[39] Xu J, Lu C, Jin S, Meng Y, Fu X, Zeng X, et al. Deep learning-based cell-specific gene regulatory networks inferred from single-cell multiome data. Nucleic Acids Res. 2025;53(5):gkaf138. 40037709 10.1093/nar/gkaf138 PMC11879466

[40] Yang K, Wang R, Liu G, Shu Z, Wang N, Zhang R, et al. HerGePred: heterogeneous network embedding representation for disease gene prediction. IEEE J Biomed Health Inform. 2018;23(4):1805–15. 10.1109/JBHI.2018.2870728 31283472

[41] Yang P, Li X-L, Mei J-P, Kwoh C-K, Ng S-K. Positive-unlabeled learning for disease gene identification. Bioinformatics. 2012;28(20):2640–7. 22923290 10.1093/bioinformatics/bts504 PMC3467748

[42] Zheng Q, Tang X, Meng Y, Xu J, Zeng X, Tian G, et al. PDGCL-DTI: parallel dual-channel graph contrastive learning for drug-target binding prediction in heterogeneous networks. IEEE J Biomed Health Infor. 2024. 10.1109/JBHI.2024.3520188. 10.1109/JBHI.2024.3520188 40030728

[43] Zhou Z, Liao Q, Wei J, Zhuo L, Wu X, Fu X, et al. Revisiting drug–protein interaction prediction: a novel global–local perspective. Bioinformatics. 2024;40(5):btae271. 38648052 10.1093/bioinformatics/btae271 PMC11087820

[44] Zhou Z, Wei J, Liu M, Zhuo L, Fu X, Zou Q. AnomalGRN: deciphering single-cell gene regulation network with graph anomaly detection. BMC Biol. 2025;23(1):73. 40069807 10.1186/s12915-025-02177-z PMC11900578

