# Supplementary material for: Denoising self-supervised learning for disease-gene association prediction
Source: BMC Bioinformatics. 2025 Oct 23;26:260. doi: 10.1186/s12859-025-06281-3 (PMC12551132; doi:10.1186/s12859-025-06281-3)
Supplement: Supplementary file 1 — Supplementary Material 1 [file 12859_2025_6281_MOESM1_ESM.pdf]

SUPPLEMENTARY INFORMATION FOR
Denoising self-supervised learning for disease-gene association prediction

Zhang et al., *BMC Bioinformatics* 2025

*Corresponding author. Email: xiang.ju@foxmail.com

Supplementary Information includes:

Supplementary Method 1.

Supplementary Table 1 to 3.

Supplementary Method 1

Modality-specific similarity construction. Taking the disease similarity computation based on drug–disease association data as an example, the process is similar to that of symptom- and GO-based similarity computation:

$$sim_{i,j}^{d,drug} = \frac{2\left|\{t_1|t_1 \in N_{d_i}\} \cap \{t_2|t_2 \in N_{d_j}\}\right|}{\left|\{t_1|t_1 \in N_{d_i}\}\right| + \left|\{t_2|t_2 \in N_{d_j}\}\right|} \tag{1}$$

where N_{d_i} and N_{d_j} represent the sets of drugs associated with d_i and d_j, respectively. Similarly, gene similarities $sim_{i,j}^{g,pathway}$, $sim_{i,j}^{g,drug}$, and $sim_{i,j}^{g,protein}$ can be obtained from pathway–protein, drug–protein, and protein–protein association data.

Similarity Fusion. By combining the previously computed disease similarity $sim_{i,j}^{d,symptom}$ and gene similarity $sim_{i,j}^{g,GO}$, we obtained multiple modality-specific similarities for diseases and genes. We then applied averaging to integrate them, thereby deriving the final disease–disease and gene–gene similarities:

$$sim_{i,j}^{d} = \frac{sim_{i,j}^{d,symptom} + sim_{i,j}^{d,drug}}{2 - a} \tag{2}$$

$$sim_{i,j}^{g} = \frac{sim_{i,j}^{g,GO} + sim_{i,j}^{g,pathway} + sim_{i,j}^{g,drug} + sim_{i,j}^{g,protein}}{4 - a} \tag{3}$$

Here, a denotes the number of modalities with zero similarity. If all modalities yield zero similarity, the similarity between the node pair is set to zero by default. This approach prevents effective similarity information from being diluted.

Relation type	Quantity	Data source
Drug-Disease	14,631	SIDER (http://sideeffects.embl.de)
Drug-Protein	277,745	STITCH (http://stitch.embl.de)
Pathway-Protein	25,813	KEGG (http://www.genome.ad.jp/kegg/)
Protein–Protein	841,068	STRING (http://string. embl.de/)

Supplementary Table 1. Information and sources of additional modalities.

Model	Evaluation Criteria						
	AUROC	AUPRC	Accuracy	Precision	Recall	F1	MCC
MiGCN	0.8693±0.001	0.8776±0.002	0.5033±0.003	0.5021±0.002	**0.9856±0.002**	0.6659±0.004	0.0225±0.002
GlaHGCL	0.8096±0.002	0.8367±0.001	0.7625±0.003	**0.9583±0.004**	0.5610±0.003	0.7077±0.002	0.5839±0.001
KDGene	<u>0.9268±0.002</u>	<u>0.9234±0.003</u>	<u>0.8283±0.002</u>	0.8900±0.003	0.7490±0.005	<u>0.8136±0.003</u>	<u>0.6650±0.004</u>
GCN	0.8414±0.003	0.8611±0.005	0.7497±0.004	0.8268±0.004	0.6304±0.004	0.7153±0.004	0.5123±0.003
VGAE	0.9211±0.001	0.9202±0.002	0.7697±0.002	0.6982±0.002	<u>0.9508±0.004</u>	0.8056±0.003	0.5761±0.003
DGSL	**0.9410±0.002***	**0.9522±0.003***	**0.8929±0.003***	<u>0.9276±0.002*</u>	0.8524±0.005	**0.8884±0.003***	**0.7885±0.006***

Supplementary Table 2. Performance comparison under 10-fold cross-validation. * indicates that DGSL significantly outperforms other methods with p-values<0.05 using the paired t-test.

Gene	Predicting scores
LMNA	0.988
GJA1	0.959
COL2A1	0.947
FGFR2	0.933
GDF5	0.932
LBR	0.931
CREBBP	0.902
HBB	0.899
FGFR3	0.896
ZMPSTE24	0.895
ATP2A2	0.893
SERPING1	0.885
EP300	0.884
FLNA	0.879
RAI1	0.856
MMP2	0.844
FIG4	0.839
WT1	0.838
PTEN	0.836
GRIP1	0.830
ATP7A	0.818
SOX9	0.812
FGFR1	0.808
PTH1R	0.800
EDA	0.797
RUNX2	0.794
PRKAR1A	0.789
VPS13A	0.787
BMPR1B	0.786
SQSTM1	0.783

Supplementary Table 3. Relevant information for the top 30 genes predicted by DGSL for Alzheimer's disease.
